# Comprehensive analysis of KAP nursing model combined with digital health management on postoperative self-management in BPH patients

**DOI:** 10.3389/fpubh.2025.1738748

**Published:** 2026-01-26

**Authors:** Changyun Chen, Xiaosi Hong, Tingting Lin, Qiongna Wang, LinLin Zhong

**Affiliations:** Urology Nursing Unit, Urology Nursing Department of the First Affiliated Hospital of Wenzhou Medical University, Wenzhou, China

**Keywords:** benign prostatic hyperplasia, digital public health, knowledge-attitude-practice model, postoperative care, quality of life, randomized controlled trial, self-management

## Abstract

**Background:**

Benign prostatic hyperplasia (BPH) is common in older men and requires surgical intervention. Optimizing postoperative recovery requires strategies to enhance patient self-management, promote healthy behaviors, and improve QoL. This study evaluated the implications of integrating behavioral nursing theory with digital public health innovation, highlighting its relevance for scalable postoperative care models in aging populations.

**Methods:**

A prospective randomized controlled trial, registered at the clinical trial registry (MR-33-2-0766-47), was conducted with 80 postoperative BPH patients undergoing transurethral resection of prostate (TURP). Patients were randomized to an intervention group (*n* = 40) receiving knowledge, attitude, and practice (KAP)-based nursing plus digital health support, or a control group (*n* = 40) receiving standard postoperative care. Outcomes (self-efficacy, illness perceptions, health-promoting behaviors, and quality of life) were assessed at baseline, 1 month, and 3 months using the General Self-Efficacy Scale (GSES), Illness Perception Questionnaire (IPQ), Health-Promoting Lifestyle Profile II (HPLP-II), and SF-36 Health Survey.

**Results:**

At 3 months post-surgery, the intervention group showed significantly higher GSES scores (32.76 ± 4.12 vs. 27.87 ± 5.12; *p* = 0.001) and HPLP-II scores (147.76 ± 8.54 vs. 137.98 ± 8.43; *p* = 0.001) compared to controls, indicating greater self-efficacy and healthier lifestyles. Illness perception improved in the intervention group, with lower IPQ “Consequences” (12.74 ± 2.87 vs. 16.52 ± 3.56; *p* = 0.002) and “Timeline” scores (11.54 ± 2.43 vs. 14.75 ± 2.65; *p* = 0.009), and higher “Control/Cure” scores (18.54 ± 4.74 vs. 14.17 ± 2.92; *p* = 0.001) than controls. SF-36 quality-of-life scores were also better in the intervention group (78.54 ± 6.76 vs. 70.32 ± 8.43; *p* = 0.001). Postoperative complication rates were lower (10% vs. 25%) and return to normal urination higher (92.5% vs. 60%) in intervention group, with significant difference in normal urination (*p* = 0.02).

**Conclusion:**

Integration of knowledge, attitude, and practice (KAP)-based nursing model with digital health management significantly improved postoperative self-efficacy, fostered healthy lifestyle practices, promoted positive illness perceptions, and enhanced overall QoL in BPH patients. This combined approach highlights the value of structured education and digital support in modern postoperative care.

**Clinical trial registration:**

https://register.clinicaltrials.gov/, identifier MR-33-2-0766-47.

## 1 Introduction

Benign prostatic hyperplasia (BPH) is a highly prevalent urological condition among men of older age. It imposes a considerable burden on quality of life (QoL) (1). BPH is characterized by prostate enlargement which leads to partial or complete urethral obstruction and lower urinary tract symptoms (LUTS) (2). According to Global Burden of Disease data, the global age-standardized prevalence of BPH in 2019 was approximately 2,480/100,000 men. This burden is expected to rise with increase in age of population (3). Moderate-to-severe BPH is managed through combination of lifestyle modifications, pharmacotherapy, and minimally invasive surgical procedures. However, the pharmacological treatments can be limited by side effects and low adherence (e.g., due to daily dose requirements) (4). Thus, the surgical intervention (transurethral resection of the prostate (TURP)) is considered as the gold standard for refractory BPH. It effectively relieves the obstruction but, on the other hand, carries the risks of complications such as bleeding, urinary incontinence, erectile dysfunction, and retrograde ejaculation. Thus, attention has optimized the postoperative management to ensure better long-term outcomes (5).

Effective self-management after TURP is crucial for recovery. Self-management is defined as active process in which patients engage in monitoring symptoms, maintain healthy lifestyles, and adhere to treatments, under the guidance of healthcare professionals (6). Self-management interventions can improve LUTS and urinary continence in BPH patients after surgery (7). The increase in postoperative self-care has been associated with decrease in complications, better control of symptom, fewer readmissions, and improvement in overall well-being of patient (8). Recent study in 2025 demonstrated that increase in self-management ability in BPH patients correlates with better well-being and quality of life of the patient (9).

The Knowledge-Attitude-Practice (KAP) model provides a framework for education and behavioral support of patient. In nursing, KAP-based approach confirms that patients receive consistent and professional guidance during stay at hospital and after discharge. The model postulates that improvement of knowledge of patient about their condition and recovery (Knowledge) will foster positive beliefs and attitudes toward management of their health (Attitude). This facilitates the adoption of healthy behaviors and adherence to care recommendations (Practice) (10). KAP interventions can reduce postoperative complications and improve knowledge of patients and self-care abilities (11). Notably, a recent study reported that continuation of nursing based on KAP theory considerably improves self-efficacy, treatment compliance, and psychological well-being in aged BPH patients (12).

In parallel to advancements in behavioral nursing models, various novelties in digital health have emerged as powerful tools in postoperative management. Digital health is made up of mobile health applications, some wearable devices, telehealth platforms, and other technologies related to health information (13). Such tools allow the monitoring in real-time, personalized education, and remote support. These all tools can collectively improve clinical outcomes across various chronic conditions such as cardiovascular disease, cancer, and BPH. In BPH, there is growing interest in usage of mobile and telemedicine technologies to support patients’ urinary symptom management and recovery (12, 14). Digital therapeutics can empower BPH patients to better self-manage voiding symptoms through personalized tools and education. Mobile health interventions allow continuous engagement beyond the hospital and can provide patients with on-demand information and feedback (15).

With this background, we hypothesized that integration of KAP nursing model with digital health management can synergistically improve postoperative self-management and outcomes in BPH patients. The study remained novel because it unites two complementary strategies, i.e., a theory-based educational framework and real-time technology-enabled support. This integration aims to enhance the knowledge of patients and motivation and also provides continuous monitoring and feedback mechanisms outside traditional clinical settings. To our knowledge, few studies have examined such a combined model in BPH postoperative care. We designed a standalone randomized controlled trial specifically for this study to assess the impact of this integrated intervention on self-efficacy, illness perceptions, health-promoting behaviors, and quality of life after TURP. This trial was not part of any broader urology research program. We also evaluated clinical recovery indicators. We predicted that patients receiving the KAP along with digital health intervention, would demonstrate superior self-management capacities and psychosocial outcomes compared to those who were given standard care. The findings of this study have broad implications for improvement postoperative recovery in BPH through the incorporation of digital innovation into nursing practice.

## 2 Materials and methods

### 2.1 Study design and participants

This single-center RCT was conducted at The First Affiliated Hospital of Wenzhou Medical University to estimate effectiveness of KAP model combined with digital health management on postoperative self-management in BPH patients. This randomized controlled trial was independently developed for the present research objectives and was not embedded within any larger departmental or institutional urology program. The trial was registered in the clinical trial registry under the identifier MR-33-2-0766-47. A total of 120 patients were assessed for eligibility. Out of them, 80 met inclusion criteria and were enrolled. The participants were all male patients (aged ≥60 years) who had undergone surgical treatment for BPH within past month. Additional inclusion criteria were: The patients having basic capability for self-management (cognitive and physical ability to participate in care). The patients were included who have ability to use smartphone or digital device (either personally or with caregiver assistance).

**Figure 1:**
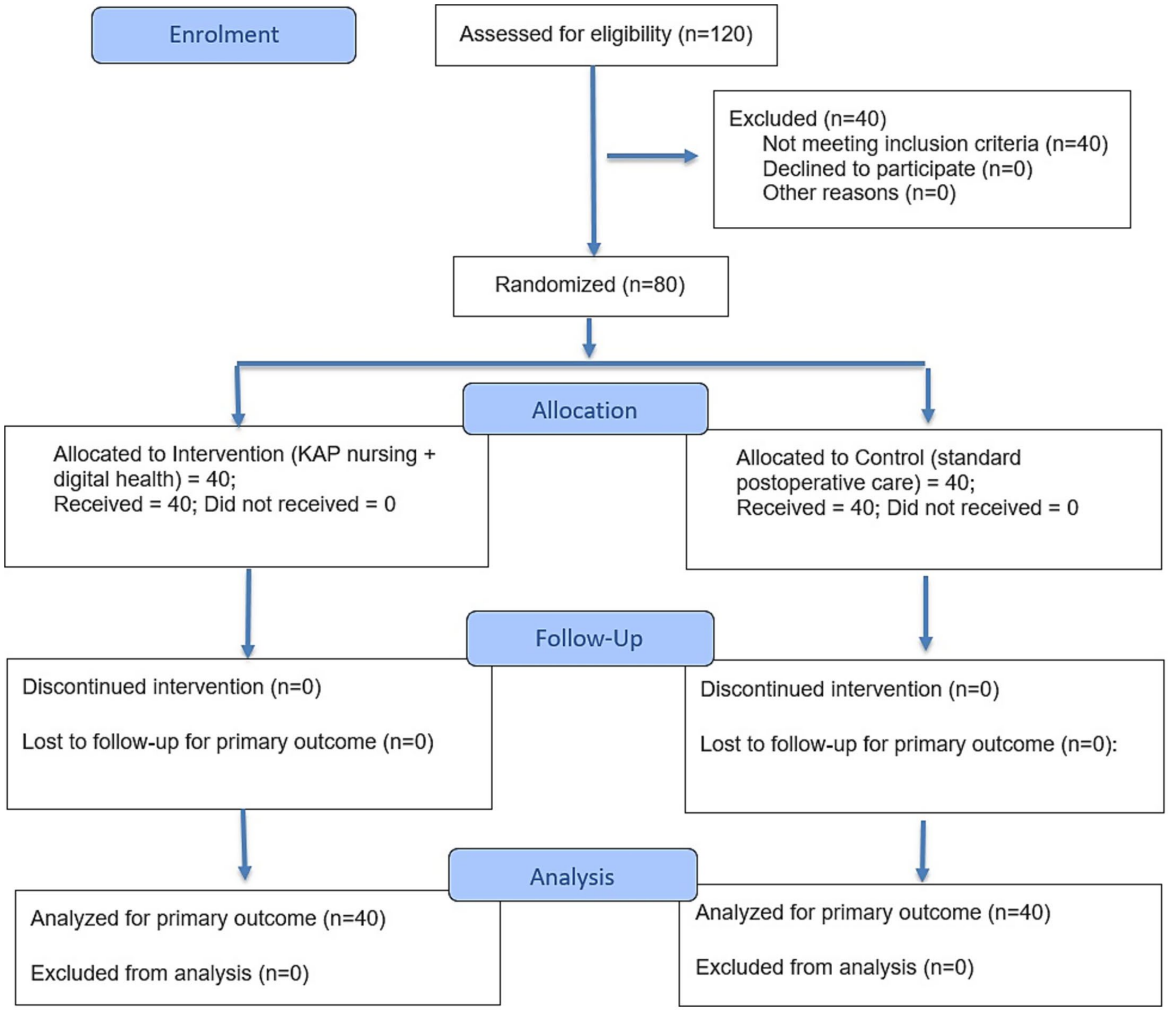
CONSORT 2025 flow diagram.

All patients were provided written informed consent to participate. The patients were excluded if patients have severe uncontrolled comorbidities or cognitive impairments. They were excluded if they are having significant post-surgical complications (e.g., recurrent urinary retention, severe infection or re-operation) at baseline. In addition, the patients were excluded if they had inability to complete follow-up (e.g., lack of contact or planning to relocate). The study followed ethical guidelines approved by Clinical Research Ethics Committee of the First Affiliated Hospital of Wenzhou Medical University (Ethical approval number: YS2023, No.286). The trial profile was illustrated using the CONSORT 2025 flow diagram as shown in Figure 1.

### 2.2 Randomization and groups

Patients were randomly assigned in a 1:1 ratio to Intervention group or Control group using a computer generated random number table. Group allocations were masked in sealed opaque envelopes which were opened after enrollment. Both groups received standard care as per hospital protocols. This included routine discharge education on postoperative expectations, catheter care when applicable, and warning signs; dietary advice to avoid bladder irritants and maintain hydration; guidance on gradually resuming activity; instruction in daily pelvic floor (Kegel) exercises; and medication management for antibiotics, analgesics, and *α*-blockers. Patients were scheduled for routine follow-up at 1 and 3 months, with additional visits as needed.

On the other hand, the intervention group received additional KAP-based nursing and digital health management measures. Due to nature of intervention, blinding of patients and care providers was not feasible. However, outcome assessors were blinded to group allocation when questionnaires were administered.

#### 2.2.1 Control group

In control group (*n* = 40), patients received standard postoperative care such as routine discharge education & follow-up. This standard care included written and verbal instructions on BPH recovery, dietary recommendations (such as a low-salt, low-fat diet and avoidance of bladder irritants like caffeine), and guidance on fluid intake, lifestyle advice, pelvic floor (Kegel) exercises, and adequate rest. This standard post-operative care also covered medication management and wound care for those with surgical incisions or catheter sites. The patients were typically seen for follow-up at 1 month and 3 months in urology clinic and with additional visits as needed. This standard care served as control condition against which the enhanced intervention was compared.

#### 2.2.2 Intervention group

In intervention group (*n* = 40), patients were given same standard care as control group plus enhanced intervention based on KAP nursing model with digital health management. In addition to routine discharge education, these patients participated in multi-domain continuous care program designed to improve their knowledge, attitudes, and practices in postoperative self-care, reinforced by digital technology:

To assess the knowledge & education (K), nursing team provided structured education through personalized one-on-one sessions once weekly (approx. 30 min each) during first month. These sessions used detailed health manuals which covers BPH management, recovery milestones, pelvic floor exercise techniques, and strategies for healthy life. Patients were also sent interactive micro-learning tasks delivered via mobile health app twice per week. Secondly, for attitude & psychological Support (A), intervention included regular counseling and encouragement. Nurses sent motivational messages three times per week through a secure messaging platform (or text message). In addition, weekly psychological counseling was provided (via phone call or online chat for approximately 15–20 min) to assess mood, address any fears or misconceptions, and promote a positive outlook toward recovery. The family members were invited to join some counseling sessions to enhance home support. To assess the practice & behavior (P), the intervention emphasized transformation of knowledge and attitude into healthy behaviors. Patients were provided with wearable smart devices that were linked to mobile app to enable real-time monitoring of key health indicators. The patients were also taught to perform daily pelvic floor exercises (Kegel exercises) and simple aerobic activities (like walking). The app also sent reminders to complete these tasks. The nursing team reviewed the incoming data weekly and used it to guide care. Nurses provided patients with a biweekly personalized progress report via app or email which contain summary of their improvements, concerns, and recommendations.

To further enhance engagement and peer support, patients in intervention group had access to online community forum which was moderated by nursing team. Through this forum, patients discussed their experiences, shared tips, and encouraged one another. Regular online community engagement and biweekly group discussions were facilitated by nurses. This community further enhanced patient engagement and self-management capacity.

### 2.3 Outcome measures

Primary outcomes were used to evaluate domains of self-management and recovery. These outcomes included self-efficacy, illness perceptions, health-promoting lifestyle behaviors, and health-related quality of life. The following validated instruments were used to assess postoperative self-management and recovery:

- General Self-Efficacy Scale (GSES) A 10-item instrument which asseses patients’ confidence in managing challenges during recovery. Scores range from 10 to 40, with higher values indicating stronger self-efficacy (16).
- Illness Perception Questionnaire (IPQ): It measures cognitive and emotional representations of illness across key domains including consequences, timeline, and control/cure. Higher consequences or timeline scores reflect more negative perceptions, whereas higher control/cure scores reflect more optimistic beliefs regarding manageability.
- Health-Promoting Lifestyle Profile II (HPLP-II): It is a 52-item scale assessing six dimensions of health-promoting behavior: nutrition, physical activity, health responsibility, stress management, interpersonal relations, and spiritual growth. Total scores range from 52 to 208, with higher scores indicating healthier lifestyle behaviors (17).
- Short Form-36 Health Survey (SF-36): It is widely used measure of health-related quality of life across eight domains of physical and mental health. Scores range from 0 to 100, with higher scores indicating better overall quality of life (18).

Secondary outcomes included clinical recovery indicators abstracted from medical records and patient self-reports. Firstly, the postoperative complications within 3 months were recorded. Secondly, we recorded the restoration of normal urination by 3 months (defined as ability to void without dysuria, with frequency <8 times/day and minimal urgency, based on patient bladder diaries and physician evaluation). Thirdly, presence of lower urinary tract symptoms (LUTS) like urgency at 3 months was noted. Patients maintained daily urinary diaries for the first month, noting voiding frequency, volumes, and any incontinence episodes or urgent symptoms, and a 3-day diary was repeated at 3 months. These objective logs, along with follow-up exam findings, were used to assess urinary outcomes.

### 2.4 Follow-up and data collection

Baseline assessments were conducted postoperatively at enrollment (just prior to intervention initiation). Follow-up assessments occurred at approximately 1 month (end of the intensive intervention period) and at 3 months. The research nurses who collected questionnaire data at follow-ups were not involved to deliver the intervention and were blinded to group allocation. For patients who could not return to hospital for follow-up, assessments were done via secure video call or phone interview and by reviewing their submitted diary records. To promote follow-up completion, reminder calls were made and transportation assistance was offered for in-clinic visits when feasible. All participants completed a 3-month follow-up period, and the overall data collection for the study was completed within a 10-month window, allowing sufficient time for enrollment, follow-up assessments, and verification of submitted records.

### 2.5 Statistical analysis

Data were analyzed by using SPSS version 29. Continuous variables were presented as mean ± standard deviation (SD). Similarly, categorical variables were presented as frequency (percentage). Baseline comparisons between the control and intervention groups were performed using independent (unpaired) Student’s *t*-tests for continuous variables and chi-square tests for categorical variables to confirm successful randomization. Because repeated measurements were obtained from the same individuals at baseline, 1 month, and 3 months, group differences over time were evaluated using a two-way repeated-measures ANOVA (Group × Time), which appropriately accounts for within-subject correlations. When Mauchly’s test indicated violation of sphericity, the Greenhouse–Geisser correction was applied. Significant interaction or main effects were followed by post-hoc pairwise comparisons between groups at each time point. To address the risk of alpha inflation due to multiple comparisons, post-hoc pairwise analyses were adjusted using the Benjamini–Hochberg false discovery rate (FDR) procedure. For outcomes assessed repeatedly over time (GSES, HPLP-II, SF-36, and IPQ domains), a two-way repeated-measures ANOVA (Group × Time) was applied, followed by FDR-corrected post-hoc comparisons where appropriate.

## 3 Results

### 3.1 Participant characteristics

Out of 120 patients screened, 80 met the inclusion criteria and were randomized (40 to each group). All of the 80 patients completed the 3-month follow-up with no dropouts. Baseline demographic and clinical characteristics of the two groups are shown in Table 1. The mean age was ~66 years in both groups. The average duration of BPH symptoms before surgery was about 10 years in each, with no significant difference. Baseline International Prostate Symptom Scores (IPSS) were similar. Crucially, there were no significant differences between the control and intervention groups in baseline GSES, total IPQ score, HPLP-II, or SF-36 scores (all *p* > 0.5). This indicated the successful randomization, as both groups were comparable in initial self-efficacy, illness perceptions, lifestyle behaviors, and quality of life prior to intervention. All post-hoc pairwise comparisons were adjusted using the Benjamini–Hochberg false discovery rate (FDR) correction to account for multiple statistical testing across repeated outcome measurements.

**Table 1:** Baseline demographic and clinical characteristics of the both groups.

Parameter	Control (*n* = 40) mean ± SD	Intervention (*n* = 40) mean ± SD	*p*-value
Age (years)	65.65 ± 5.43	66.42 ± 4.12	0.72^NS^
Duration of BPH (years)	10.12 ± 3.87	9.82 ± 4.87	0.36^NS^
IPSS (symptom score)	20.23 ± 4.54	19.78 ± 5.93	0.54^NS^
GSES (self-efficacy)	23.18 ± 4.24	23.92 ± 4.39	0.76^NS^
IPQ total (illness perception)	61.48 ± 7.12	60.93 ± 6.32	0.69^NS^
HPLP-II (lifestyle)	128.54 ± 7.34	129.28 ± 6.45	0.81^NS^
SF-36 (quality of life)	64.32 ± 5.76	65.43 ± 5.12	0.77^NS^

NS, not significant (p > 0.05). Baseline scores compared by independent student’s *t*-test; IPSS, international prostate symptom score; HPLP-II, Health-Promoting Lifestyle Profile-II; SF-36; Short Form Health Survey-36.

### 3.2 Self-efficacy significantly improved in the intervention group

**Figure 2:**
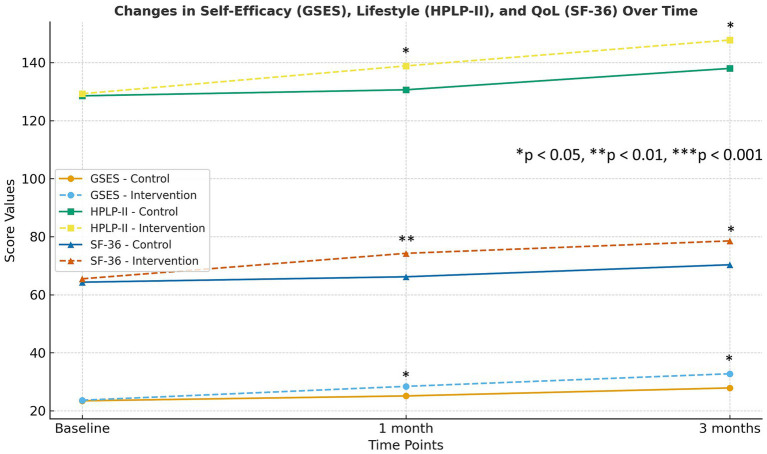
Trends in mean scores for the general self-efficacy scale (GSES), Health-Promoting Lifestyle Profile II (HPLP-II), and Short Form-36 (SF-36) at baseline, 1 month, and 3 months postoperatively. Error bars represent mean ± SD. Asterisks denote between-group differences at each time point: ^*^p < 0.05, ^**^p < 0.01, ^***^p < 0.001.

The intervention group demonstrated a significantly greater increase in GSES scores over time compared with the control group. Repeated-measures ANOVA showed a significant Group × Time interaction for GSES scores (*p* < 0.001), indicating that changes in self-efficacy over time differed significantly between groups. Baseline GSES scores (around 23–24 out of 40) indicated moderate self-efficacy in both groups and did not differ significantly (Table 2, Figure 2). After 1 month, the intervention group’s mean GSES had increased to 28.42 ± 3.42, significantly higher than control group’s 25.12 ± 3.65 (*p* = 0.001). This improvement in the intervention arm became even more pronounced by 3 months. The intervention group reached a mean GSES of 32.76 ± 4.12 vs. 27.87 ± 5.12 in controls (*p* = 0.001). Thus, over time the KAP + digital intervention group exhibited a robust gain in self-efficacy, whereas the control group showed only a modest increase. The between-group differences at both follow-ups were highly significant. Importantly, 90% of patients in intervention group achieved a GSES score ≥30 (indicating high confidence) by 3 months, compared to only 55% in the control group.

**Table 2:** Comparison of GSES, HPLP-II, Short Form (SF)-36 health survey, Illness Perception Questionnaire domain scores among the groups at different follow-up periods.

Time point	Control (*n* = 40) mean ± SD	Intervention (*n* = 40) mean ± SD	*p*-value (group difference)
GSES scores			
Baseline	23.44 ± 4.26	23.65 ± 5.12	0.84^NS^
1 month	25.12 ± 3.65	28.42 ± 3.42	0.001^*^
3 months	27.87 ± 5.12	32.76 ± 4.12	0.001^*^
Health-Promoting Lifestyle Profile II (HPLP-II) scores			
Baseline	128.54 ± 7.65	129.27 ± 6.42	0.65^NS^
1 month	130.62 ± 8.46	138.85 ± 9.43	0.007^*^
3 months	137.98 ± 8.43	147.76 ± 8.54	0.001^*^
Short Form (SF)-36 health survey scores			
Baseline	64.32 ± 5.65	65.48 ± 5.87	0.65^NS^
1 month	66.18 ± 6.76	74.26 ± 6.45	0.001^*^
3 months	70.32 ± 8.43	78.54 ± 6.76	0.001^*^
Illness Perception Questionnaire domain scores			
Consequences			
Baseline	18.43 ± 3.21	18.13 ± 2.92	0.65^NS^
1 month	17.65 ± 4.32	14.82 ± 2.54	0.001^*^
3 month	16.52 ± 3.56	12.74 ± 2.87	0.002^*^
Timeline			
Baseline	16.25 ± 3.76	16.54 ± 2.87	0.74^NS^
1 month	15.92 ± 3.12	12.18 ± 2.24	0.001^*^
3 month	14.75 ± 2.65	11.54 ± 2.43	0.009^*^
Control/cure			
Baseline	12.54 ± 2.87	12.32 ± 2.65	0.42^NS^
1 month	13.47 ± 3.65	16.72 ± 2.98	0.003^*^
3 month	14.17 ± 2.92	18.54 ± 4.74	0.001^*^

GSES range 10–40 (higher = better self-efficacy). HPLP-II total score range 52–208 (higher = more health-promoting behaviors). SF-36 total score range 0–100 (higher = better QoL). *p* by unpaired *t*-test. Values are group means. Higher “Consequences”/“Timeline” scores = more negative perception; higher “Control/Cure” = more positive perception. ^*^indicates *p* < 0.05. NS, not significant.

### 3.3 Intervention group adopted healthier lifestyle behaviors

Participants receiving the KAP-based digital intervention showed markedly larger improvements in overall HPLP-II lifestyle scores than those receiving standard care. Repeated-measures ANOVA demonstrated a significant Group × Time interaction for HPLP-II scores (*p* < 0.001), with the intervention group showing greater improvements in lifestyle behaviors across follow-up. At baseline, the overall HPLP-II scores indicated moderate engagement in health-promoting behaviors (approx. 129 out of 208 in both groups, with no significant difference). Over time, the intervention group adopted significantly healthier lifestyles than controls (Table 2 and Figure 2). After 1 month, the HPLP-II score of intervention group rose to 138.85 ± 9.43 vs. 130.62 ± 8.46 in controls (*p* = 0.007). By 3 months, this gap increase with intervention group averaged at 147.76 ± 8.54 as compared to 137.98 ± 8.43 in controls (*p* = 0.001). Notably, the intervention group improved in all subdomains of the HPLP-II, with largest gains was observed in physical activity and health responsibility. For instance, 80% of intervention patients reported exercising at least 3 times per week by month 3, versus about 50% of controls. Dietary habits also improved; more patients in intervention adopted recommended diets consistently. The control group showed some improvement, but it was markedly smaller.

### 3.4 Quality of life improved more substantially in the intervention group

SF-36 scores increased significantly more in the intervention group than in the control group, indicating broader gains in postoperative well-being. Repeated-measures ANOVA revealed a significant Group × Time interaction for SF-36 scores (*p* < 0.001), indicating larger QoL improvements in the intervention group compared with controls. Health-related quality of life improved in both groups after surgery. However there was more significant improvement in intervention group. Baseline SF-36 total scores (~64 to 65) reflected the impact of BPH symptoms on well-being of participants prior to intervention. By 1 month, SF-36 score of intervention group increased to 74.26 ± 6.45, compared to 66.18 ± 6.76 in controls (*p* = 0.001). By 3 months, intervention group achieved mean SF-36 of 78.54 ± 6.76, which surpassed SF-36 of control group, i.e., 70.32 ± 8.43 (*p* = 0.001), as shown in Table 2 and Figure 2. This indicated a broad enhancement in quality of life domains for intervention patients, approaching levels seen in healthy older adults. In contrast, control patients still lagged behind in overall recovery of well-being. The domains of physical functioning and role limitations (physical) showed particularly large between-group differences. By 3 months, 85% of intervention patients reported no limitation in moderate activities (like walking a few blocks or carrying groceries), versus 60% of controls. Mental health and vitality scores were also higher in intervention group, which showed the improvement in illness perceptions and reduction in anxiety.

### 3.5 Illness perceptions became more positive in the intervention group

**Figure 3:**
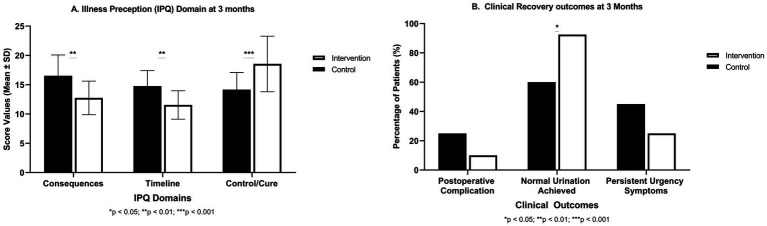
**(A)** shows group differences in Illness Perception Questionnaire (IPQ) domains (Consequences, Timeline, and Control/Cure) with mean ± SD values. **(B)** compares clinical recovery outcomes (postoperative complications, normal urination, and persistent urgency), demonstrating improved physiological and functional recovery under the KAP + Digital Health program. Bars show mean ± SD for IPQ domains and percentages for clinical outcomes. Statistical significance between groups is indicated by asterisks above the bars: ^*^p < 0.05, ^**^p < 0.01, ^***^p < 0.001.

We examined illness perceptions of patients by using key IPQ domains (Consequences, Timeline, Control/Cure). Repeated-measures ANOVA demonstrated significant Group × Time interactions across the IPQ Consequences (*p* < 0.01), Timeline (*p* < 0.01), and Control/Cure (*p* < 0.001) domains, indicating that changes in illness perceptions differed substantially between groups over time. Compared with controls, intervention participants showed greater reductions in negative illness perceptions and larger increases in perceived control/cure. At baseline, both groups held comparable insights about BPH. They moderately acknowledged its consequences and chronic nature, and had similar beliefs in controllability (Table 2, Figure 3A, baseline rows; all *p* > 0.5). After intervention, significant divergences emerged. In consequences domain, this reflects the perceived severity and life impact of condition. At 1 month, the Consequences score of intervention group dropped to 14.82 ± 2.54 from 18.13 at baseline. It indicated that patients viewed impact of BPH as less severe. Meanwhile, the control group remained higher at 17.65 ± 4.32. The difference was significant (*p* = 0.001). By 3 months, the score of intervention group further improved to 12.74 ± 2.87, versus 16.52 ± 3.56 in controls (*p* = 0.002). Lower scores in this domain suggest intervention patients felt their condition was better managed and less debilitating over time. In timeline domain, this represents how long-term or chronicity of the patients. At 1 month, intervention patients had lower Timeline score (12.18 ± 2.24) than controls (15.92 ± 3.12; *p* = 0.001), and similarly at 3 months (11.54 ± 2.43 vs. 14.75 ± 2.65; *p* = 0.009). Thus, the intervention group moved showed illness as less prolonged or more likely to be resolved, compared to the control group who continued to see it as a longer-term issue. In Control/Cure domain, the degree of optimism was measured about controlling or curing the condition. Higher scores are better here. After 1 month, intervention group reported significantly greater perceived control (16.72 ± 2.98) than controls (13.47 ± 3.65; *p* = 0.003). By 3 months, this gap increased and showed 18.54 ± 4.74 in the intervention vs. 14.17 ± 2.92 in controls (*p* = 0.001). Intervention patients grew more confident that their actions and treatment could manage or cure their condition.

### 3.6 Faster urinary recovery and fewer complications observed in the intervention group

In addition to psychosocial measures, we tracked concrete clinical outcomes up to 3 months (Table 3, Figure 3B). The incidence of postoperative complications (primarily mild urinary tract infections and transient urinary retention episodes) was lower in the intervention group (4 patients, 10%) as compared to the control group (10 patients, 25%). Although this difference did not reach conventional statistical significance (*χ*^2^ = 2.16, *p* = 0.14) but still it suggested a positive trend. Notably, all complications were minor and managed conservatively. There were no re-hospitalizations for BPH-related issues in intervention group and two in control group (one for urinary retention which requires catheterization, one for severe LUTS which requires medication adjustment).

**Table 3:** Clinical recovery outcomes by 3 months.

Outcome	Control (*n* = 40)	Intervention (*n* = 40)	*p* (Chi-square)
Any postoperative complication^†^	10 (25%)	4 (10%)	0.14^NS^
Normal urination achieved	24 (60%)	37 (92.5%)	0.02^*^
Persistent urgency symptoms	18 (45%)	10 (25%)	0.10^NS^

Minor complications (e.g., UTI, retention) managed conservatively. *p* by chi-square test; NS, not significant. * indicates *p* < 0.05 and is significant.

A significant difference was found in rate of return to normal urination by 3 months. In the intervention group, 37 out of 40 patients (92.5%) had resumed normal voiding patterns, compared to 24/40 (60%) in control group (*χ*^2^ = 10.65, *p* = 0.02). Patients in the intervention group reported fewer persistent LUTS. Only 25% reported urgency symptoms at 3 months, versus 45% in controls. Although, this difference was not statistically significant (*p* = 0.10). The better urinary outcomes in the intervention arm coincide with higher adherence to pelvic floor exercises and earlier reporting of symptoms (due to close monitoring), which allowed timely interventions (like bladder training advice or medication where appropriate).

## 4 Discussion

This randomized controlled trial examined a new integrated solution that involves KAP-based nursing model and digital health management to enhance self-management in BPH patients after surgery. Our results indicate that this co-intervention made significant improvement in self-efficacy, health promotion behaviors, illness perceptions and QoL of patients compared to standard care. At 3 months post-surgery, the intervention group reported better results in all the domains measured. The patients felt more confident and assertive in the health management, their lifestyles were healthier, their attitude toward their disease was more optimistic, and their physical and mental well-being was better. There were also indications of improved clinical recovery. These conclusions favor effectiveness of incorporation of behavioral nursing models into digital health tools in order to optimize postoperative care.

To our knowledge, this study is the first in BPH population to use the KAP educational model in conjunction with telehealth and mHealth technologies in the application of postoperative care. This broad approach is consistent with larger movement toward individualized, patient-centered practice of public health. Continuous monitoring and support were made possible through the use of digital health management. It also serves as an effective extension of health care team beyond the hospital. Patients in intervention group could receive timely feedback and interventions (19). This echoes the idea of technology as a complement to hospital to home transition, which is the essence of recent healthcare developments (20). Particularly, our digital platform facilitated real-time tracking of each recovery progress of patient (urination patterns, activity levels, etc.) and two-way communication between patients and providers.

Self-efficacy was main central target and outcome of our intervention group. The significant improvement in GSES scores in the intervention group highlights that program effectively empowered patients. Self-efficacy is widely accepted as essential factor of adherence and long-term self-care success. Patients with higher confidence in managing their condition are more likely to follow through on exercise, diet, and symptom monitoring routines (21). Our approach combined the consistent education, encouragement, and evidence of personal progress via data feedback which likely reinforced self-belief of patients. We observed that as self-efficacy increased, the patients took more initiative, which created positive feedback loop. These results are supported by other studies in recent past. Mao et al. reported that continuous nursing intervention (grounded in KAP theory) led to higher self-efficacy and better compliance in BPH patients (12). Likewise, Peng et al. demonstrated that mobile health program enhanced self-care agency of post intraprostatic surgery patients. Our work extends this knowledge by demonstrating that the interventions related to nursing and technological can have an even greater influence on self-efficacy when combined (19).

The improvement in health-promoting behaviors (HPLP-II) highlights the practical lifestyle impact of intervention. In our research, we obtained a high level of improvement of the lifestyle in intervention group through application of regular digital reminders and individual follow-up. Patients experienced better diets, physical exercises, and management of stress. Initial education and motivation was offered with the help of the KAP model and translated into everyday practice with the help of the digital platform. The long-term persistence of these changes is encouraging toward long-term habit-forming formation. This is especially true in the view of the public health. The impact of encouraging health lifestyles among older adults has spillover effects and prevents the risk of comorbid diseases such as disease of cardiovascular system or diabetes (22). Our intervention influenced the lifestyle behaviors which are in line with evidence from digital health research. As an example, Behr et al. examined digital program based health promoting behavior, which demonstrated the +0.11 improvement of overall HPLP-II scores in the 6 months intervention (23). Our trial was shorter but achieved comparable magnitude of change in lifestyle metrics. Our study suggested that even a 3-month targeted intervention can initiate meaningful health behavior changes in this population. Such results indicate that incorporation of micro learning components, inspirational text messaging, and consistent feedback via digital technology facilitated long term behavioral effects. These findings align with health promotion theories and previous studies that indicated effectiveness of customized technology-assisted interventions (24).

Quality of life is ultimate measure of success for many interventions, especially in a benign condition like BPH (5). In this study, patients in the intervention group experienced significantly greater improvements in SF-36 quality-of-life scores compared with those receiving standard care. These gains were observed across both physical and mental health domains, reflecting better postoperative recovery, increased confidence in managing symptoms, and enhanced daily functioning. The magnitude of improvement—approaching normative values for older adults by 3 months—underscores the added value of integrating KAP-based education with digital health tools to support recovery after TURP (19).

Correspondingly, the intervention patients had more favorable illness perceptions. By provision of knowledge and demonstration of progress, we helped patients to perceive BPH as manageable condition rather than life-defining chronic disease. Lower perceived consequences and timeline scores in intervention group suggest reduced illness related uneasiness and sense that normal life can resume. In addition, higher control beliefs indicated that those patients felt their own actions and treatment could effectively control BPH. A recent cross-sectional study found that better self-management abilities and lower anxiety correlated with improved subjective well-being in BPH patients. This study emphasized the linkage of psychological and behavioral factors (9). Similar to our study, a mobile or web self-care platform for management of benign and malignant diseases showed significant improvements in brief IPQ control/cure domain scores (+2.8 points; *p* < 0.001) and emotional representation domain after 3 months of intervention (23). The present study findings indicate that the digital health interventions positively influenced cognitive and emotional representation of illness. It further highlighted greater optimism, perceived control, and adaptive management in BPH patients (14).

From a clinical view, one of most tangible benefits observed was the faster recovery of normal urination in intervention group. This has direct relevance to BPH outcomes because faster alleviation of LUTS after surgery is considered as key indicator of success (25). Indeed, recent study on telehealth in urology noted that integrated telehealth approaches can improve outcomes while potentially causes the reduction in costs and utilization of resources (26). Our findings are in line with this study. The patients with enhanced follow-up had fewer emergency visits and unplanned calls than typical for our clinic’s TURP patients, which suggested more efficient care management.

This study has several limitations that should be considered when interpreting the findings. First of all, the sample size is relatively small (*n* = 80) and is recruited in only one center and in a limited geographical area, which limits the external validity of the results. Second, the nature of the intervention did not allow the blinding of the subjects and care providers; hence, presenting the possibility of bias in the performance; this was partly offset by the use of blinded assessors in questionnaire administration. Thirdly, the sole use of self-reported measurements created a possible reporting bias, which was mitigated by using a subset of the results to verify some device-based data (e.g., accelerometer readings, to confirm exercise diaries). However, there were still chances of reporting falsehoods, especially with regards to compliance and personal perceived well-being. Although multiple outcomes were assessed across several time points, we mitigated the risk of type I error by applying the Benjamini–Hochberg FDR correction, which improves the reliability of the observed statistical differences. The future research could be strengthened by the use of objective biomarkers, including the results of activity-trackers or biochemical endpoints. Lastly, the current research did not involve direct evaluation of digital literacy in the participants, future research should evaluate the possibility of adjusting the intensity of interventions based on the technological literacy of patients.

## 5 Conclusion

In conclusion, integration of knowledge, attitude, and practice (KAP)-based nursing model with digital health management produced significant improvements in self-efficacy, lifestyle behaviors, illness perceptions, quality of life, and urinary recovery among patients undergoing TURP. By combining structured education with continuous digital support, the intervention strengthened patient engagement and postoperative self-management. These findings suggest promising opportunities for clinicians to adopt blended behavioral–digital strategies to enhance routine urological care. For policymakers and hospital administrators, the model offers a scalable and resource-efficient framework that may reduce complications, support aging populations, and improve long-term recovery outcomes. Future studies should evaluate cost-effectiveness, assess implementation across diverse healthcare settings, and explore how digital literacy and technology access influence program adoption and impact.

## Data Availability

The raw data supporting the conclusions of this article will be made available by the authors, without undue reservation.
